# Elevated Levels of Serum Neurofilament Light Chain Associated with Cognitive Impairment in Vascular Dementia

**DOI:** 10.1155/2020/6612871

**Published:** 2020-11-01

**Authors:** Weibin Ma, Jingjing Zhang, Jialei Xu, Depeng Feng, Xiaoling Wang, Fengyu Zhang

**Affiliations:** Department of Neurology, Liaocheng People's Hospital, Liaocheng, Shandong, China 252000

## Abstract

**Objective:**

Vascular dementia (VaD) is a progressive neurodegenerative disease with cognitive decline caused by cerebrovascular factors. Despite the great progress made in the past decade, VaD still lacks effective treatments and peripheral blood biomarkers. In this study, we tested the level of peripheral blood neurofilament light chain (NfL) in VaD patients and explored its relationship with cognitive impairment.

**Method:**

A total of 176 study subjects including 80 normal controls (NC) and 96 VaD patients were included in our study. Upon admission, we collected clinical and biochemical characteristics of all research subjects. We also evaluate the Montreal cognitive assessment scale (MoCA) scores of all subjects. The serum NfL level was measured by the single-molecule array (Simoa) method.

**Results:**

The years of education in the NC group and VaD group were (11.65 ± 3.04) years and (10.53 ± 3.87) years, respectively. Compared with VaD patients, the NC group has a higher level of education (*p* = 0.037). Furthermore, the results of Simoa indicated that VaD subjects had higher serum NfL levels compared with the NC group [(8.49 ± 2.37) pg/ml vs. (19.26 ± 4.71) pg/ml, *p* < 0.001]. In terms of other clinical and biochemical characteristics, there was no significant difference between VaD and NC. The Spearman correlation analysis indicated that educational years have a significant positive correlation with MoCA scores (*r* = 0.238, *p* = 0.041), while age and serum NfL levels have a significantly negative correlation with MoCA scores (age: *r* = −0.213, *p* = 0.040; NfL: *r* = −0.395, *p* = 0.027). However, further multiple regression analysis showed that only serum NfL level might serve as an independent risk factor for cognitive decline in VaD (*β* = 0.317, *p* = 0.021).

**Conclusion:**

The serum NfL levels in VaD subjects are significantly elevated, which may be used as a potential peripheral blood marker for predicting cognitive impairment in patients with VaD.

## 1. Introduction

Vascular dementia (VaD) can be defined as a neurodegenerative disease related to vascular brain injury, which is mainly manifested as cognitive decline and memory loss [1, 2]. With the advent of a graying society, the number of people suffering from dementia has increased substantially. According to reports, the number of people diagnosed with dementia will double every 20 years [3]. Therefore, the number of dementia patients will reach 66 million in 2030 and 120 million in 2050 [4, 5]. VaD accounts for about 15%-20% of all dementias, and its incidence is second only to Alzheimer's disease (AD) [6]. Unlike AD, there is no licensed treatment for vascular dementia. In addition, VaD also lacks effective therapeutic targets [7, 8]. Therefore, it is becoming imminent to find reliable molecular biomarkers to assess the potential risk of VaD.

Neurofilaments (Nf) is a cytoskeletal protein mainly expressed in neurons, which belongs to Type IV intermediate filament family having structural similarity with some protein molecules such as nestin, peripherin, and a-internexin [9]. According to their molecular weight, Nf is biologically divided into three subunits: 68 kDa Nf light (NfL), 160 kDa Nf medium (NfM), and 205 kDa Nf heavy (NfH) chains [10]. Recently, Nf is considered as a biomarker of neuroaxonal damage. When brain parenchyma is damaged, Nf is released into cerebrospinal fluid (CSF) and peripheral blood circulation [11, 12]. The traditional enzyme-linked immunosorbent assay can only detect Nf in CSF, but the more accurate single-molecule array technology makes it possible to detect Nf in peripheral blood [13, 14].

Among the subunits of Nf, NfL is among the most promising biomarker candidates [15]. There is mounting evidence that NfL is related to a series of neurodegenerative diseases [16]. However, the relationship between NfL and VaD is still dim. The purpose of this research is to detect the serum level of NfL and to further clarify whether NfL can be used as a potential biomarker for the prevention and treatment in VaD.

## 2. Patients and Methods

### 2.1. Patient Population

The current study is a prospective cross-sectional descriptive design. Totally, 96 patients with VaD and 80 normal controls who were admitted to Liaocheng People's Hospital from June 2018 to May 2020 were recruited. Diagnosis of VaD was confirmed by attending neurologist according to the National Institute for Neurological Disorders and Stroke (NINDS-AIREN) and Diagnostic and Statistical Manual of Mental Disorders (DSM-V) [17, 18]. Exclusion criteria were as follows: (1) suffering from other types of dementia; (2) suffering from mental illness; (3) suffering from acute cerebrovascular disease or infectious disease; (4) malignant tumor; (5) history of surgery or other severe trauma within 3 months; (6) heart, liver, or kidney insufficiency and other serious acute and chronic diseases. This research abided by the Declaration of Helsinki. We obtained written consent from all subjects and approved by the Ethics Committee of Liaocheng People's Hospital.

### 2.2. Clinical and Biochemical Characteristics

At admission, clinical and biochemical characteristics were collected: education years, smoking and alcohol habits, coronary heart disease (CHD), high blood pressure (HBP), systolic blood pressure (SBP), diastolic blood pressure (DBP), hyperlipidemia (HLP), total cholesterol (TC), triglycerides (TG), high-density lipoprotein cholesterol (HDL-C), low-density lipoprotein cholesterol (LDL-C), Diabetes Mellitus (DM), and fasting plasma glucose (FBG).

### 2.3. Cognitive Function Testing

Montreal cognitive assessment (MoCA) tool is a popular cognitive assessment tool having high sensitivity and specificity [19]. MoCA evaluation indicators include the following: alternating trail making, visuoconstructional skills, naming, memory, visuoconstructional skills attention, sentence repetition, verbal fluency, abstraction, delayed recall, and orientation. The MoCA evaluation takes about 10 minutes, and the total score is 30 points. One point is added to subjects who have received formal education for less than 12 years. A final total score of 26 and above is considered normal. The MoCA cognitive assessment is conducted under standard conditions by a professionally trained neurologist who is blind to the subjects' clinical baseline data.

### 2.4. Measurement of Serum NfL Levels

All subjects collected fasting serum samples within 24 hours of admission and stored them at −80°C. The serum levels of NfL were tested by the single-molecule array method (Simoa). The blood sample was placed at room temperature for 15 minutes and then centrifuged at 4°C for 15 minutes at a speed of 2000 × g to obtain serum. The Simoa NF-light assay used commercial reagents (UmanDiagnostics, Umea, Sweden) on an HD-1 platform (Neoline, Hangzhou, China) according to manufacturer's instructions. All samples were tested blindly, and the measurement was repeated [20].

### 2.5. Statistical Analysis

The concentration of NfL in serum showed a normal distribution. In this study, categorical variables are recorded by numbers (percentage, %), while continuous variables are recorded by mean ± standard deviation (mean ± SD). The *t*-test was applied for the comparison of continuous variables, and the chi-square test was applied for the comparison of categorical variables. Spearman's correlation analysis is used to assess the binary correlation. Multivariate regression analysis was applied to assess the predictive value of clinical and biochemical characteristics on the cognitive function in patients with VaD. The SPSS 22.0 statistical software (SPSS Inc., IL, USA) was used in the study, and a *p* value of 0.05 was considered significant.

## 3. Results

### 3.1. Clinical and Biochemical Characteristics

**Figure 1:**
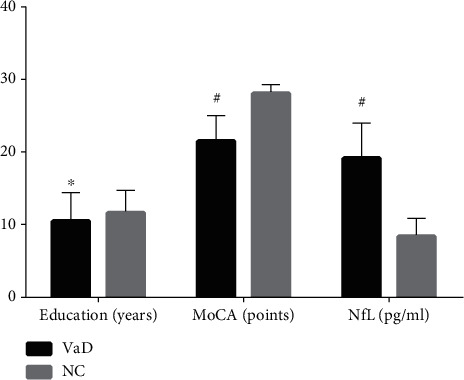
The differences in education years, MoCA scores, and serum NfL levels between VaD and NC. MoCA: Montreal cognitive assessment scale; NfL: neurofilament light chain; VaD: vascular dementia; NC: normal controls. Compared with NC group, ^∗^*p* = 0.037, ^#^*p* < 0.001.

A total of 176 subjects including 96 VaD patients and 80 normal controls admitted to Liaocheng People's Hospital from June 2018 to May 2020 were enrolled. The clinical and biochemical characteristics of all subjects were presented in Table 1. No significant differences were found in age, gender, smoking and alcohol habits, history of chronic disease (CHD, HBP, HLP, and DM), SBP, DBP, TC, TG, HDL-C, LDL-C, and FBG between VaD and NC. However, significant differences were found in education years, MoCA scores, and serum NfL levels between the two groups (Figure 1).

### 3.2. Spearman's Correlation Analysis

The correlation between clinical and biochemical characteristics and MoCA score was assessed by Spearman's correlation analysis. The results of the correlation analysis are presented in Table 2. The results indicated that age (*r* = −0.213, *p* = 0.040) and the levels of serum NfL (*r* = −0.395, *p* = 0.027) were negatively correlated with MoCA scores in VaD patients, and the correlation was significant. The results also indicated that that the education years in VaD is positively correlated with the MoCA score (*r* = 0.238, *p* = 0.041). However, in our current study, there is no significant correlation between cognitive decline and other clinical and biochemical characteristics of VaD patients (*p* > 0.05).

### 3.3. Multiple Regression Analysis

The results of multiple regression analysis of MoCA score and serum NfL level in VaD patients are presented in Table 3. The results showed that serum NfL level may serve as an independent predictive risk factor for the cognitive decline in patients with VaD. After adjusting for age, gender, years of education, SBP, DBP, TC, TG, HDL-C, LDL-C, FBG, and other clinical and biochemical characteristics, the serum NfL level still has important significance for the independent value of cognitive function in VaD (*β* = 0.317, *p* = 0.021).

## 4. Discussion

The aim of the research was to study the relationship between cognitive function and serum NfL levels in VaD patients as well as normal controls. The results showed that the serum NfL level in VaD patients was significantly higher than that in the normal control group, while the MoCA score was lower. We also found that the MoCA score in VaD was significantly negatively correlated with serum NfL levels and age and positively correlated with years of education but had no significant correlation with other clinical and biochemical characteristics. This association is not affected by disturbing factors such as age, gender, years of education, SBP, DBP, TC, TG, HDL-C, LDL-C, and FBG. To our knowledge, we have confirmed for the first time that serum NfL may serve as an independent risk factor for cognitive impairment in patients with VaD.

It has recently been discovered that NfL is related to a battery of neurological diseases. Johanna Gaiottino and his colleagues found that patients with AD, amyotrophic lateral sclerosis (ALS), and Guillain-Barré-syndrome (GBS) have higher levels of cerebrospinal fluid and serum NfL, and this change in NfL level is not accompanied by evidence of structural damage of central nervous system (CNS) [21]. Ching-Hua Lu's team further confirmed that serum NfL level was an easily accessible marker for prognostic of patients with ALS [22]. Interestingly, NfL mutations can cause severe early onset of Charcot–Marie–Tooth (CMT) disease [23]. In addition, elevated levels of NfL in cerebrospinal fluid or peripheral blood have also been reported in Parkinson's disease, relapsing-remitting multiple sclerosis, progressive supranuclear palsy, and brain metastases [24–28]. All the above studies suggested that NfL can be used as a biological target of certain nervous system disorders.

Emerging evidence indicates that NfL is involved in the pathological mechanism of cognitive declines [29]. A Chinese Taipei study showed that plasma NfL is a biomarker of cognitive decline in AD and Parkinson's disease (PD), and it is more specific for AD [24]. A 5-year longitudinal retrospective study in the United States showed that higher serum NfL levels are related to poor current and future clinical and cognitive performance [30]. In hereditary frontotemporal dementia, blood NfL can be used as a biomarker of disease progression, and the longitudinal measurement of NfL provides valuable information as a marker of treatment effect [31]. Not only in frontotemporal dementia, a Swedish study showed that plasma NfL is a noninvasive biomarker for AD [32]. Although the relationship between NfL and cognitive impairment has been widely reported, its mechanism is not completely clear.

Our research has some limitations. Firstly, the current study is a single-center study with a small sample size. Secondly, we did not monitor the serum NfL level longitudinally nor did we dynamically follow up the cognitive function and prognosis of patients. Thirdly, because the gold standard for diagnosis of VaD is biopsy, thus, our diagnosis for VaD may not be accurate enough, and VaD patients may also accompany by other types of cognitive impairment. However, our study confirmed for the first time the correlation between elevated serum NfL levels and cognitive decline, which has important clinical potential application value in VaD.

## 5. Conclusion

In conclusion, our study mainly found that the serum NfL levels of VaD patients were significantly elevated than that of the NC group. Our study reported for the first time that serum NfL plays an important role in the pathophysiology of the cognitive function of VaD patients. We expect the emergence of larger multicenter studies to confirm the association between serum NfL concentrations and cognitive function in patients with VaD. Elucidating the potential pathological mechanism that NfL involved in the pathogenesis of VaD will have great applied value.

## Figures and Tables

**Table 1:** Clinical and biochemical characteristics of all the subjects.

Variables	NC	VaD	*p*
*N*	80	96	—
Age (years)	69.59 ± 5.31	69.27 ± 6.18	0.742
Gender (male/female)	49/31	60/36	0.865
Serum NfL (pg/mL)	8.49 ± 2.37	19.26 ± 4.71	<0.001
Education (years)	11.65 ± 3.04	10.53 ± 3.87	0.037
Smoking (*n*, %)	19 (23.75)	24 (25.00)	0.848
Alcohol (*n*, %)	27 (33.75)	30 (31.25)	0.724
CHD (*n*, %)	10 (12.50)	13 (13.54)	0.838
HBP (*n*, %)	24 (30.00)	29 (30.21)	0.976
SBP (mmHg)	134.46 ± 14.28	135.82 ± 13.59	0.519
DBP (mmHg)	87.62 ± 10.35	88.17 ± 11.63	0.743
HLP (*n*, %)	17 (21.25)	20 (20.83)	0.946
TC (mmol/L)	4.72 ± 0.93	4.89 ± 1.14	0.286
TG (mmol/L)	1.64 ± 0.31	1.69 ± 0.52	0.451
HDL-C (mmol/L)	1.26 ± 0.39	1.21 ± 0.28	0.325
LDL-C (mmol/L)	2.41 ± 0.67	2.59 ± 0.75	0.098
DM (*n*, %)	14 (17.50)	16 (16.67)	0.884
FBG (mmol/L)	6.59 ± 1.16	6.77 ± 1.38	0.356
MoCA (points)	28.12 ± 1.08	21.57 ± 3.40	<0.001

NC: normal controls; VaD: vascular dementia; NfL: neurofilament light chain; CHD: coronary heart disease; HBP: high blood pressure; SBP: systolic blood pressure; DBP: diastolic blood pressure; HLP: hyperlipidemia; TC: total cholesterol; TG: triglycerides; HDL-C: high-density lipoprotein cholesterol; LDL-C: low-density lipoprotein cholesterol; DM: Diabetes Mellitus; FBG: fasting plasma glucose; MoCA: Montreal cognitive assessment scale.

**Table 2:** Correlation coefficients between MoCA scores and various parameters in VaD.

	MoCA (points)	
	*r*	*p*
Age (years)	-0.213	0.040
Gender	0.301	0.454
Education (years)	0.238	0.041
SBP (mmHg)	-0.372	0.245
DBP (mmHg)	-0.526	0.418
TG (mmol/L)	-0.377	0.183
TC (mmol/L)	-0.429	0.204
HDL-C (mmol/L)	0.284	0.312
LDL-C (mmol/L)	-0.267	0.059
FBG (mmol/L)	-0.482	0.656
Serum NfL (pg/mL)	-0.395	0.027

MoCA: Montreal cognitive assessment scale; VaD: vascular dementia; SBP: systolic blood pressure; DBP: diastolic blood pressure; TC: total cholesterol; TG: triglycerides; HDL-C: high-density lipoprotein cholesterol; LDL-C: low-density lipoprotein cholesterol; FBG: fasting plasma glucose; NfL: neurofilament light chain.

**Table 3:** Association between MoCA scores and various parameters in VaD.

	Regression coefficient	*p*	95% CI
Age (years)	0.138	0.184	0.725-1.203
Gender	0.263	0.196	0.788-1.194
Education (years)	0.214	0.086	0.672 - 1.145
SBP (mmHg)	0.386	0.437	0.783 - 1.092
DBP (mmHg)	0.379	0.322	0.561 - 1.236
TG (mmol/L)	0.272	0.195	0.826 - 1.215
TC (mmol/L)	0.305	0.618	0.487 - 1.309
HDL-C (mmol/L)	0.403	0.239	0.799 - 1.167
LDL-C (mmol/L)	0.260	0.193	0.915 - 1.128
FBG (mmol/L)	0.621	0.264	0.871 - 1.053
Serum NfL (pg/mL)	0.317	0.021	1.634 - 2.481

MoCA: Montreal cognitive assessment scale; VaD: vascular dementia; SBP: systolic blood pressure; DBP: diastolic blood pressure; TC: total cholesterol; TG: triglycerides; HDL-C: high-density lipoprotein cholesterol; LDL-C: low-density lipoprotein cholesterol; FBG: fasting plasma glucose; NfL: neurofilament light chain.

## Data Availability

The data used to support the findings of this study are available from the corresponding author upon request.
